# Design and Evaluation of a Novel Variable Stiffness Hip Joint Exoskeleton

**DOI:** 10.3390/s24206693

**Published:** 2024-10-17

**Authors:** Tao Yang, Chifu Yang, Feng Jiang, Bowen Tian

**Affiliations:** 1 School of Mechatronics Engineering, Harbin Institute of Technology, Harbin 150001, China; 20b908071@stu.hit.edu.cn (T.Y.); cfyang@hit.edu.cn (C.Y.); 2 School of Computer Science and Technology, Harbin Institute of Technology, Harbin 150001, China; 3 School of Business Administration, Zhongnan University of Economics and Law, Wuhan 430000, China; callytian1128@alumni.hust.edu.cn

**Keywords:** exoskeleton, antagonistic actuation, variable stiffness mechanism, coordinated control of torque and stiffness

## Abstract

An exoskeleton is a wearable device with human–machine interaction characteristics. An ideal exoskeleton should have kinematic and kinetic characteristics similar to those of the wearer. Most traditional exoskeletons are driven by rigid actuators based on joint torque or position control algorithms. In order to achieve better human–robot interaction, flexible actuators have been introduced into exoskeletons. However, exoskeletons with fixed stiffness cannot adapt to changing stiffness requirements during assistance. In order to achieve collaborative control of stiffness and torque, a bionic variable stiffness hip joint exoskeleton (BVS-HJE) is designed in this article. The exoskeleton proposed in this article is inspired by the muscles that come in agonist–antagonist pairs, whose actuators are arranged in an antagonistic form on both sides of the hip joint. Compared with other exoskeletons, it has antagonistic actuators with variable stiffness mechanisms, which allow the stiffness control of the exoskeleton joint independent of force (or position) control. A BVS-HJE model was established to study its variable stiffness and static characteristics. Based on the characteristics of the BVS-HJE, a control strategy is proposed that can achieve independent adjustment of joint torque and joint stiffness. In addition, the variable stiffness mechanism can estimate the output force based on the established mathematical model through an encoder, thus eliminating the additional force sensors in the control process. Finally, the variable stiffness properties of the actuator and the controllability of joint stiffness and joint torque were verified through experiments.

## 1. Introduction

Many processes in industry still require manual work, although the level of automation has improved. Some tasks that require lifting and carrying loads and squatting for manual handling work may pose a risk to the health of workers. Among these manual tasks, squatting during load-lifting operations is a significant hazard to the health of workers due to the high muscle load, fast exercise frequency, and lack of effective rest [1]. These risk factors have cumulative effects and mainly lead to lower back pain and musculoskeletal disorders of the lower limbs [2,3]. When the fatigue level of the quadriceps femoris in the legs is high, the load on the lower back also increases, which significantly increases the risk of lower back injury [4]. This type of work-related injury usually causes significant discomfort to employees and may even lead to early withdrawal from work or affect normal physiological activities. As wearable devices, exoskeletons can be used not only for rehabilitation training during medical treatment but also to enhance the strength of workers in the industry. In recent years, different forms of lower limb and upper limb exoskeletons have been developed [5,6,7,8,9,10].

At present, traditional exoskeletons are typically driven by rigid actuators, which adopt joint torque or position control algorithms [11,12,13]. These algorithms are realized by controlling the motor rotation based on the difference between the actual trajectory and the expected trajectory, resulting in their implementation being relatively simple [14]. Both position and torque control algorithms belong to the trajectory tracking algorithm of single closed-loop control. Traditional rigid actuators provide exoskeletons with excellent trajectory-tracking capabilities, enabling them to achieve effective assistance in relatively constant environments. However, the human–robot interaction environment is diverse and unpredictable, and traditional rigid actuators, which rely on joint torque or position control algorithms, cannot adapt well to the dynamic laws of interaction with the environment, leading to unnatural and rigid performance [15]. As a result, this high rigidity is likely to cause harm to users [16]. The safety and nature of human–robot interactions are particularly important when using exoskeletons to assist the human body [17]. Therefore, some scholars have begun to pay attention to human–computer interaction issues. A new model considering human-exoskeleton coupling dynamics has been proposed, which can be used to evaluate human performance and the exoskeleton’s assistance [18]. The new model is helpful for designing comfortable human–computer interaction. To guarantee the operator’s safety in human-exoskeleton cooperative motion, the output constraint control of lower limb exoskeletons has been proposed in [19]. This research constructs a probability model of joint motion, obtains a time-varying joint-position-constrained boundary based on sparse Gaussian processes, and utilizes a backstepping controller with a finite time extended state observer to compensate for uncertainty.

In order to solve the problems of human–robot interactions, compliance is introduced into the exoskeleton. The methods for achieving joint compliance can be divided into active and passive types. There are no elastic components in active compliance actuators, but control algorithms are designed to satisfy specific relationships between the output force and position. Thus, a rigid actuator is simulated to exhibit the behavior of a spring. A typical example of active flexible control is impedance control, which has been widely applied to exoskeletons [20,21,22]. The advantage of an active compliant actuator is that it can change the presenting stiffness characteristics by modifying the control algorithm parameters, thereby achieving online adjustment of the actuator’s flexibility [23,24]. However, due to the lack of elastic components, active compliant actuators cannot store energy and absorb impacts, which poses a potential risk of injury to users [25]. Passive compliance control typically involves artificially adding passive flexible components such as springs to the motion chain of the actuator [26]. The aim is to replace the contact stiffness at the original parameter position with a low stiffness known to the elastic body parameters, utilizing the passive characteristics of elastic components that can absorb or store energy and thereby reducing the structural stiffness of the rigid actuator. The series elastic actuator (SEA) is the most common type of passive compliant actuator, and researchers have applied the SEA and its variants to the exoskeleton system [27,28,29]. However, due to the stiffness of such SEA-based passive flexible actuators depending on the stiffness parameters of the series elastic components, they do not have the ability to vary stiffness and cannot be adjusted online during joint driving. In fact, organisms have the ability to regulate the impedance of limb joints to maintain stability in uncertain environments. For example, the stiffness of lower limb joints in humans constantly changes throughout the entire gait cycle. Therefore, researchers have placed more emphasis on variable stiffness actuators.

The existing methods of variable stiffness adjustment mainly include the following: elastic structure control, mechanical control, and antagonistic drive control. Elastic structure control achieves variable stiffness by changing the effective number of turns and the effective segment length of the elastic body [30,31]. Although this method has a simple basis and convenient control, the range of stiffness is very limited and is influenced by the spring material and configuration. For mechanical control, stiffness adjustment is realized by changing the preload of the elastic body or the transmission ratio at the output end [32,33,34,35]. This method usually requires one motor to adjust the position and another to adjust the stiffness separately. The inspiration for antagonistic control comes from bionics, where actuators are typically arranged on both sides of the driving joint [36,37]. This requires complex structural design and increases the control difficulty in order to achieve nonlinear stiffness in the spring. However, the physiological mechanisms of the human body have not been considered for control, and stiffness modulation lacks biomimetic design.

The safety of the human exoskeleton interaction process is particularly important, and the sensing and controllability of assistant forces/torques are given priority consideration. The most common method is to use torque sensors or force sensors [38]. However, high-resolution torque sensors are expensive, and they are typically installed at joints, which increases the rotational inertia of exoskeletons. A method for estimating and controlling torque without using expensive torque sensors is proposed in [39]. This method relies on joint stiffness estimation and is prone to errors. A torque-estimation method combining encoder and tension sensor information is used in [40]. This method is simple and effective, but the tension sensor and spring are connected in series and arranged outside the variable stiffness unit, which increases the overall size. Considering that the variable stiffness actuator in this study has a rotating structure, a torque-estimation and control method that only requires encoders as sensors is presented. Human motion information is collected by encoders at the joints and compensated by servo motors. The torque of the exoskeleton is estimated through the encoder at the variable stiffness unit, which avoids the use of expensive torque sensors. In addition, sensors integrated in variable stiffness units have smaller motion inertia compared to those installed in exoskeleton joints.

Each joint is controlled by a pair of agonist–antagonist muscles, which have nonlinear impedance characteristics. When the agonist–antagonist muscle groups contract, the stiffness of the driven joint will change. This antagonistic arrangement of muscles enables the human body to independently control joint movement and joint stiffness [41]. We aimed to develop a bionic variable stiffness hip joint exoskeleton (BVS-HJE) and conduct feasibility experiments on a prototype. The main contributions of this paper include the following: (1) A cable-driven antagonistic hip exoskeleton with variable stiffness units is designed. (2) The mathematical model of BVS-HJE is established, and a torque and stiffness estimation method that adopts the encoder as the signal source is presented. (3) A control strategy is proposed that can achieve independent adjustment of joint torque and joint stiffness.

The structure of this letter is as follows. The mechanical structure and corresponding mathematical models of the BVS-HJE are established in Section 2. A stiffness-estimation model for the hip joint and a decoupling control algorithm for joint torque and stiffness are proposed in Section 3. In Section 4, the performance of the BVS-HJE is evaluated experimentally. The work is concluded in Section 5.

## 2. Mechanical and Mathematical Models of the BVS-HJE

### 2.1. Hip Joint Exoskeleton Mechanism

**Figure 1:**
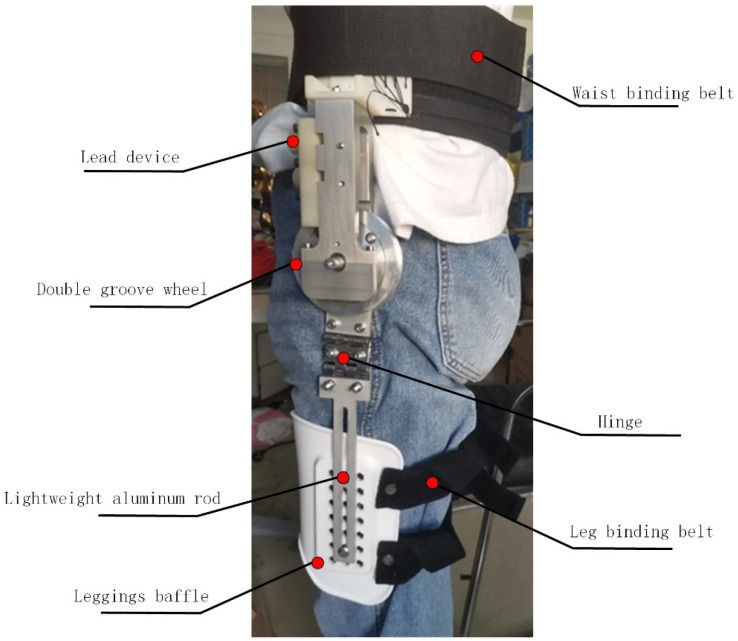
Schematic diagram of the hip joint exoskeleton mechanism.

The mechanical structure of the hip joint is shown in Figure 1, consisting of a binding belt, lead device, double groove wheel, hinge, lightweight aluminum rod, and leggings baffle. The binding belt connected to the waist is made with thick fabric, ensuring it is lightweight and improving the fit to the human body during wear. The lead device is used to introduce the Bowden cables into the exoskeleton, serving as a constraint and connection. There are two constraint pulleys placed inside, each responsible for guiding the Bowden cables on the flexion and extension sides of the hip. The Bowden cables on the hip flexion and extension sides are all bound to the double-grooved wheel, controlling the rotation of the double-grooved wheel in the sagittal plane. The hinge mechanism can better fit the surface of the human thigh, meeting the passive degrees of freedom of hip joint movement. The inner part of the leggings baffle is embedded with flexible fabric to improve the fit and comfort of the wearer’s limb surface. At the same time, the relative position of the baffle on the lightweight aluminum rod can be adjusted to adapt to the length of the human leg.

Considering that some operators have different motion styles and features, the exoskeletons are designed with passively adjustable connections to adapt to different individual features. Firstly, the fixed position of the legs can be adjusted steplessly. The relative position of the baffle on the lightweight aluminum rod can be adjusted according to the wearer’s preferences by changing the position of the fixing screws. Then, the double hinges connected between the exoskeleton hip joint and the lightweight aluminum rod can provide the wearer with passive degrees of freedom. The focus of this study is the rotation in the sagittal plane, while the passive degrees of freedom provided by hinges in the coronal plane can make exoskeletons more compliant with the human body.

### 2.2. Variable Stiffness Mechanism

The variable stiffness mechanisms of the hip flexion and extension sides are the same, and both transmit tension through the Bowden cables. Therefore, in this article, only the principle of one side is introduced.

**Figure 2:**
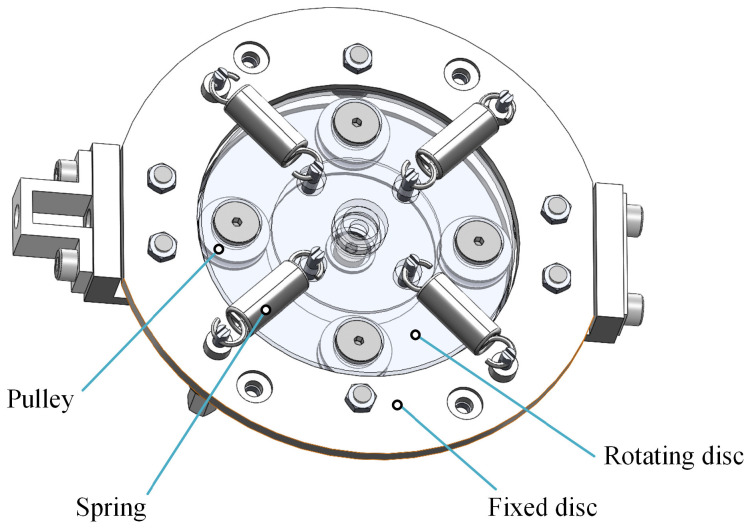
Schematic diagram of the variable stiffness mechanism.

The rotary variable stiffness device proposed in this article and driven by the cable is shown in Figure 2, consisting of a fixed disc, a rotating disc, four springs, and several pulleys. A pulley block is installed on one side of the rotating disc, and the other side of the rotating disc is connected to four sets of springs. Under the action of the inner cable, the rotating disc generates rotational motion relative to the fixed disc while pulling the spring to generate torque. The stiffness of the mechanism varies at different rotation angles.

### 2.3. Mathematical Modeling of the BVS-HJE

**Figure 3:**
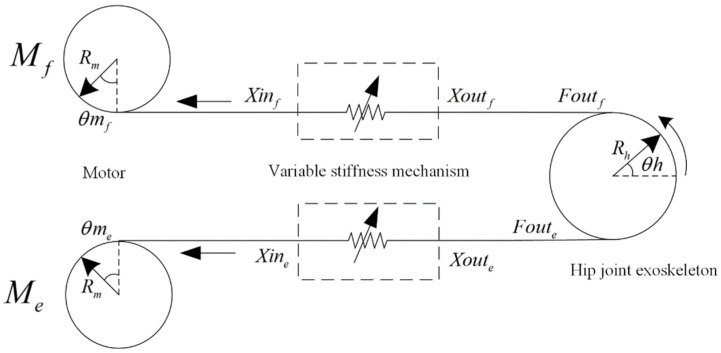
Schematic diagram of the BVS-HJE.

This article follows the principle of antagonistic muscles to drive the exoskeleton from the side of hip flexion and extension, respectively. A schematic diagram of the BVS-HJE is shown in Figure 3, where the subscripts f and e represent the hip flexion and extension sides, respectively. The subscripts in the following text are all defined in this way. $M_{f}$ represents the motor on the flexion side; $R_{m}$ is the effective radius of the winding wheel of the motor; $Xin_{f}$ and $Xout_{f}$ are the displacements of the input and output ends of the variable stiffness mechanism on the side of hip flexion, respectively; $Fout_{f}$ is its output force. $R_{h}$ and θh are the radius and angle of the exoskeleton joint, respectively.

According to Figure 3, a kinematic model of the BVS-HJE can be obtained as follows:

(1) $$\left\{\begin{array}{l} \Delta L_{f}=Xin_{f}-Xout_{f}\\ Xin_{f}=R_{m}\cdot \theta m_{f}\\ Xout_{f}=R_{h}\cdot \theta h\\ \Delta L_{e}=Xin_{e}-Xout_{e}\\ Xin_{e}=R_{m}\cdot \theta m_{e}\\ Xout_{e}=-R_{h}\cdot \theta h \end{array}\right.,$$

where $\Delta L_{f}$ and $\Delta L_{e}$ are the deformation variables of the variable stiffness mechanism on the hip flexion and extension sides, respectively. The output torque $Tout_{E}$ and stiffness $Kout_{E}$ of the hip joint exoskeleton can be expressed as

(2) $$\left\{\begin{array}{l} Tout_{E}=R_{h}\cdot \left(Fout_{f}-Fout_{e}\right)\\ \begin{array}{ccc} Kout_{E} & = & R_{h}\cdot \left(\frac{dFout_{f}}{-d\theta h}+\frac{dFout_{e}}{d\theta h}\right)\\ & = & R_{h}\cdot \left(-\frac{dFout_{f}}{d\Delta L_{f}}\frac{d\Delta L_{f}}{d\theta h}+\frac{dFout_{e}}{d\Delta L_{e}}\frac{d\Delta L_{e}}{d\theta h}\right)\\ & = & {R_{h}}^{2}\cdot \left(K_{f}+K_{e}\right) \end{array}\\ K_{f}=\frac{dFout_{f}}{d\Delta L_{f}},\;K_{e}=\frac{dFout_{e}}{d\Delta L_{e}} \end{array}\right.,$$

where $K_{f}$ and $K_{e}$ are the equivalent stiffness of the variable stiffness mechanism on the hip flexion and extension sides, respectively.

**Figure 4:**
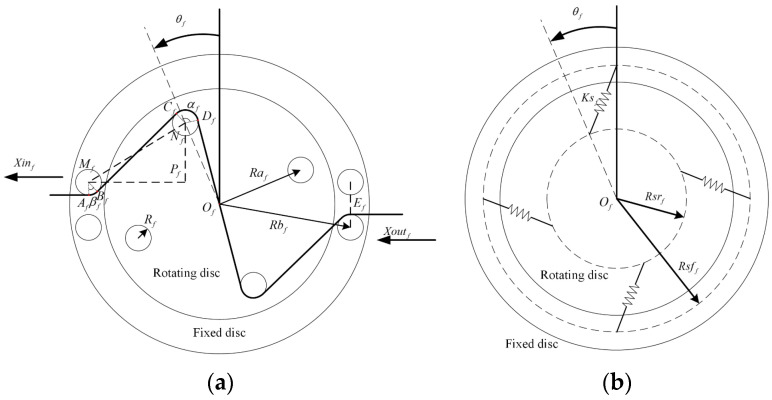
Simplified model of the variable stiffness mechanism. (**a**) The side with pulley blocks; (**b**) the side with springs.

The simplified model of the variable stiffness mechanism is shown in Figure 4. For ease of explanation, we only introduce the hip flexion side. $Xin_{f}$ and $Xout_{f}$ represent the displacement of the input and output cables, respectively. $\theta_{f}$ is the rotation angle of the inner plate. All pulleys have the same radius and are represented by $R_{f}$. $Ra_{f}$ is the distribution radius of the pulleys on the rotating disc. $Rb_{f}$ is the distribution radius of the pulleys on the fixed plate. $E_{f}$ is the distance between two adjacent pulleys on the fixed disc. $\alpha_{f}$ and $\beta_{f}$ refer to the wrap angle of the cable to one pulley on the rotating and fixed discs, respectively. The distribution radius of the spring hooks on the rotating disc is $Rsr_{f}$, and that of the fixed disc is $Rsf_{f}$. The stiffness of each spring is $Ks_{f}$.

According to the geometric relationship in Figure 4, the relationship between the effective length of the inner cable and the rotation angle in the variable stiffness mechanism is

(3) $$\left\{\begin{array}{l} L_{f}(\theta_{f})=2\cdot (A_{f}B_{f}+C_{f}D_{f}+B_{f}C_{f}+D_{f}O_{f})\\ A_{f}B_{f}+C_{f}D_{f}=R_{f}\left(\alpha_{f}+\beta_{f}\right)\\ B_{f}C_{f}=2\sqrt{{\left(\frac{M_{f}N_{f}}{2}\right)}^{2}-{R_{f}}^{2}},\;D_{f}O_{f}=\sqrt{R{a_{f}}^{2}-{R_{f}}^{2}}\\ M_{f}{N_{f}}^{2}=M_{f}{P_{f}}^{2}+N_{f}{P_{f}}^{2}\\ M_{f}P_{f}=\sqrt{R{b_{f}}^{2}-{\left(\frac{E_{f}}{2}\right)}^{2}}-Ra_{f}\sin\left(\theta_{f}\right)\\ N_{f}P_{f}=Ra_{f}\cos\left(\theta_{f}\right)-\frac{E_{f}}{2}\\ \alpha_{f}=2\pi -a\cos\left(\frac{2R_{f}}{M_{f}N_{f}}\right)-\mathrm{atan}(\frac{M_{f}P_{f}}{N_{f}P_{f}})-\theta_{f}-a\cos\left(\frac{R_{f}}{Ra_{f}}\right)\\ \beta_{f}=\pi -\mathrm{acos}(\frac{2R_{f}}{M_{f}N_{f}})-\mathrm{atan}(\frac{M_{f}P_{f}}{N_{f}P_{f}}) \end{array}\right.,$$

The deformation variable $\Delta L_{f}$ caused by the rotation angle of the mechanism changing from $\theta_{f1}$ to $\theta_{f2}$ can be expressed as

(4) $$\Delta L_{f}=L_{f}(\theta_{f1})-L_{f}(\theta_{f2}),$$

The recovery torque $Ts_{f}$ generated by the four springs on the rotating disc is

(5) $$\left\{\begin{array}{l} Ts_{f}=4\cdot Rsr_{f}\cdot \left[Ks_{f}\cdot \left(L-Rsf_{f}+Rsr_{f}\right)+F_{0}\right]\cdot \left[\frac{Rsf_{f}}{L}\sin\left(\theta_{f}\right)\right]\\ L=\sqrt{Rs{f_{f}}^{2}+Rs{r_{f}}^{2}-2\cdot Rsf_{f}\cdot Rsr_{f}\cdot \cos\left(\theta_{f}\right)} \end{array}\right.,$$

**Figure 5:**
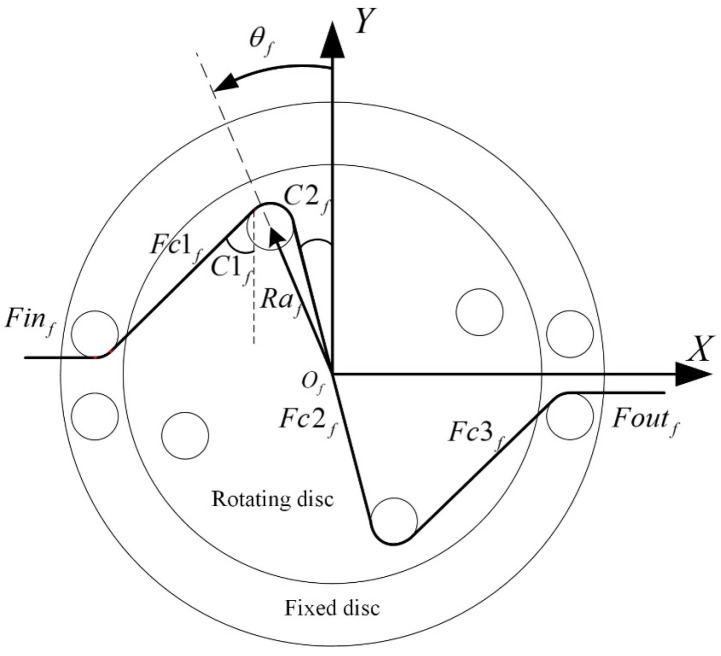
Schematic diagram of the stress situation for the inner cable.

Then, in order to facilitate analysis of the effect of the cable, the cable is divided into different sections. From the input side, they are marked as $Fin_{f}$, $Fc1_{f}$, $Fc2_{f}$, $Fc3_{f}$, and $Fout_{f}$, respectively. A schematic diagram of the cable forces is shown in Figure 5 The internal structure of the mechanism is centrally symmetrical; therefore, the geometric parameters of only one pulley were analyzed.

The torque generated by each cable force on the central rotating plant can be expressed as

(6) $$\left\{\begin{array}{l} Tc1_{f}=Ra_{f}\cdot Fc1_{f}\cdot \sin\left(C1_{f}+\theta_{f}\right)\\ Tc2_{f}=Ra_{f}\cdot Fc2_{f}\cdot \sin\left(\theta_{f}-C2_{f}\right)\\ Tc3_{f}=Ra_{f}\cdot Fc3_{f}\cdot \sin\left(C1_{f}+\theta_{f}\right)\\ C1_{f}=a\mathrm{atan}(\frac{M_{f}P_{f}}{N_{f}P_{f}})-a\sin\left(\frac{2R_{f}}{M_{f}N_{f}}\right),\;C2_{f}=\theta_{f}-a\sin\left(\frac{R_{f}}{Ra_{f}}\right) \end{array}\right.,$$

According to Formula (6), we can obtain the traction torque $Tc_{f}$ of the inner cable on the rotating plant as

(7) $$Tc_{f}=Tc1_{f}+2Tc2_{f}+Tc3_{f},$$

According to mechanical balance, the recovery torque of the spring and the traction torque of the rope should be equal. In the ideal case of no friction, the output force $Fout_{f}$ is equal to the tension force $Fci_{f}$ of each section of the rope. However, there is friction between the pulley and the cable, and the output force $Fout_{f}$ and tension force $Fci_{f}$ of each section of the cable are not equal. To make the modeling more accurate, friction compensation is required. Considering that friction models based on the static Coulomb friction model are widely used in research and are highly practical, the Coulomb friction model was adopted in this article. The static equilibrium relationship of a tiny displacement segment $\left[x,x+dx\right]$ of the cable on the pulley satisfies

(8) $$\left\{\begin{array}{l} F\left(x+dx\right)=F\left(x\right)+\mathrm{sgn}\left(\dot{x}\right)f\left(x\right)\\ f\left(x\right)=\mu N\left(x\right),\;N\left(x\right)=\frac{F\left(x\right)}{R}dx \end{array}\right.,$$

where $F\left(x\right)$, $f\left(x\right)$, and $N\left(x\right)$ are the tension, friction, and positive pressure of the cable, respectively; μ and R are the friction coefficient and pulley radius, respectively; and $\mathrm{sgn}\left(\dot{x}\right)$ represents the direction of motion of the cable.

In order to facilitate analysis of the relationship between each section of the cable, Formula (8) is transformed into an exponential expression:

(9) $$\begin{array}{ccc} F\left(x+dx\right) & = & F\left(x\right)\left[1+\frac{\mathrm{sgn}\left(\dot{x}\right)\mu }{R}dx\right]\\ & = & F\left(x\right)\exp\left[\frac{\mathrm{sgn}\left(\dot{x}\right)\mu }{R}dx\right] \end{array},$$

By integrating Formula (9), the relationship between the forces at both ends of the cable wrapped on the pulley can be obtained as

(10) $$F\left(D\right)=F\left(C\right)\exp\left[\mathrm{sgn}\left(\dot{x}\right)\mu \alpha \right],$$

where C and D are the two ends of the cable on the pulley, respectively, and α is the pulley wrap angle from C to D.

By combining Formulas (6), (7), and (10), we can obtain the relationship between the output force $Fout_{f}$ of the mechanism and the traction torque generated by the cable, which is

(11) $$\left\{\begin{array}{l} Tc_{f}=Ra_{f}\cdot \left[Fc1_{f}\cdot \sin\left(C1_{f}+\theta_{f}\right)+2\cdot Fc2_{f}\cdot \sin\left(\theta_{f}-C2_{f}\right)+Fc3_{f}\cdot \sin\left(C1_{f}+\theta_{f}\right)\right]\\ Fc3_{f}=Fout_{f}\exp\left[\mathrm{sgn}\left(\dot{x}\right)\mu \beta_{f}\right]\\ Fc2_{f}=Fout_{f}\exp\left[\mathrm{sgn}\left(\dot{x}\right)\mu \left(\alpha_{f}+\beta_{f}\right)\right]\\ Fc1_{f}=Fout_{f}\exp\left[\mathrm{sgn}\left(\dot{x}\right)\mu \left(2\alpha_{f}+\beta_{f}\right)\right] \end{array}\right.,$$

where the directional function $\mathrm{sgn}\left(\dot{x}\right)$ can be rewritten as

(12) $$\mathrm{sgn}\left(\dot{x}\right)=\left\{\begin{array}{ll} 1 & \mathrm{loading}\\ 0 & \mathrm{motionless}\\ -1 & \mathrm{unloading} \end{array}\right.,$$

According to mechanical balance, the recovery torque of the spring and the traction torque of the rope should be equal. We thus obtain

(13) $$Tc_{f}=Ts_{f}=4\cdot Rsr_{f}\cdot \left[Ks_{f}\cdot \left(L-Rsf_{f}+Rsr_{f}\right)+F_{0}\right]\cdot \left[\frac{Rsf_{f}}{L}\sin\left(\theta_{f}\right)\right],$$

By combining Formulas (11) and (13), we can obtain the output force $Fout_{f}$ of the mechanism at the angle $\theta_{f}$. According to the relationship between the deformation variable of the cable and the angle of rotation in Formula (3), the equivalent stiffness $K_{f}$ of one variable stiffness mechanism can be expressed as

(14) $$K_{f}=\frac{dFout_{f}}{d\Delta L_{f}}=\frac{dFout_{f}}{d\theta_{f}}/\frac{d\Delta L_{f}}{d\theta_{f}},$$

In Formulas (11) and (14), it can be seen that the equivalent stiffness $K_{f}$ and output force $Fout_{f}$ of the mechanism depend on the rotation angle $\theta_{f}$—that is, they are coupled. Therefore, it is difficult for one variable stiffness mechanism to simultaneously control the output force and stiffness. Human joints are synergistically controlled by antagonistic muscle groups, and when they produce varying degrees of contraction, joint stiffness and torque can be simultaneously controlled. The BVS-HJE proposed in this article also adopts this bilateral antagonistic driving method. According to Formula (2), it can be inferred that the output torque is controlled by the output force of the variable stiffness mechanism on both sides in a common mode form and the output stiffness is controlled in a differential form by the equivalent stiffness of the variable stiffness mechanism on both sides, and they are all determined by the rotation angle of the variable stiffness mechanism. Therefore, we can obtain the expected torque and stiffness of the exoskeleton simultaneously by controlling the rotation angles of the two motors.

Considering that the size of the double-grooved wheel at the hip joint is not abrupt relative to the human body, its radius $R_{h}$ is designed to be 0.04 m. In order to be as compact as possible, the radius $R_{m}$ of the drive motor is designed to be 0.02 m, and small pulleys with the radius R of 0.007 m are used in the variable stiffness unit. Considering that two small pulleys cannot interfere with each other, the minimum distance between the centers of the two pulleys is 0.014 m. To leave a certain margin, the distance E between two adjacent pulleys is designed to be 0.018 m. The maximum movement angle of the human hip joint is 2.5 rad, so the maximum displacement of the Bowden cable at the hip joint groove wheel is 0.1 m. To meet the requirement that the variable stiffness mechanism can generate a maximum displacement of 0.1 m at a maximum angle of 1.57 rad, combined with formula 3, the distribution radius Ra of the pulleys on the rotating disc is 0.027 m, and the distribution radius Rb of the pulleys on the fixed plate is 0.045 m. Correspondingly, the distribution radius Rsf of spring hooks on the other side of the fixed disk can also be designed as 0.045 m. To ensure non-interference, the distribution radius Rsr of spring hooks on the rotating disk is set to 0.015 m. The mechanical parameters of the device in this article are shown in Table 1.

## 3. Controller Design

In the previous section, an estimation model for the output torque and stiffness of one variable stiffness mechanism was established based on the rotation angle. A variable stiffness mechanism in this article is similar to the traditional series elastic actuator, and an accurate estimation of the output force can be obtained by detecting the deformation of the elastic body. Meanwhile, the output of the hip exoskeleton is coordinately controlled by the variable stiffness mechanisms on both the flexion and extension sides.

For reference torque $T_{ref}$ and stiffness $K_{ref}$ at a certain joint angle θh, coordinated control can be achieved by simultaneously adjusting the rotation angle of the flexion and extension motor. According to the torque relationship in Formula (2), the output force of the variable stiffness mechanism on both sides of the hip joint should satisfy

(15) $$T_{ref}=Tout_{E}=R_{h}\cdot \left(Fout_{f}-Fout_{e}\right),$$

where $Fout_{f}$ is considered as an independent variable, and then $Fout_{e}$ can be expressed as

(16) $$Fout_{e}=Fout_{f}-\frac{T_{ref}}{R_{h}},$$

According to Formula (16), when the output force of the variable stiffness mechanism of the extension side is determined, the output force of the flexion side is also determined accordingly. Formulas (11) and (13) describe the relationship between the output force of the variable stiffness mechanism and the rotation angle of the mechanism. This relationship can be rewritten as

(17) $$\left\{\begin{array}{l} Fout_{f}=f\left(\theta_{f}\right)\\ Fout_{e}=f\left(\theta_{e}\right) \end{array}\right.,$$

where f is the function of Fout about θ, which is equivalent to Formula (13). Then, the function of $\theta_{f}$ about $Fout_{f}$ can be obtained as

(18) $$\left\{\begin{array}{l} \theta_{f}=f^{-1}\left(Fout_{f}\right)\\ \theta_{e}=f^{-1}\left(Fout_{e}\right) \end{array}\right.,$$

where $f^{-1}$ is the inverse function of f. f is an explicit function, and its inverse function $f^{-1}$ is an implicit function. It is difficult to solve the implicit function, so polynomials were used to fit $f^{-1}$ in this article.

The relationship between the stiffness and rotation angle of the mechanism is described in Formula (14). Combining Formulas (14) and (18), the output stiffness can be expressed by the output force, which can be abbreviated as

(19) $$\left\{\begin{array}{l} K_{f}=g\left(\theta_{f}\right)=g\left[f^{-1}\left(Fout_{f}\right)\right]=h\left(Fout_{f}\right)\\ K_{e}=g\left(\theta_{e}\right)=g\left[f^{-1}\left(Fout_{e}\right)\right]=h\left(Fout_{e}\right) \end{array}\right.,$$

where g is the function of K about θ, which is equivalent to Formula (14), and h is the function of K about Fout, which can be obtained by introducing the inverse function $f^{-1}$ into the function g. Combining Formula (19) and the stiffness relationship in Formula (2), the output stiffness of the variable stiffness mechanism on both sides of the hip joint should meet

(20) $$\begin{array}{ccc} K_{ref} & = & Kout_{E}={R_{h}}^{2}\cdot \left(K_{f}+K_{e}\right)\\ & = & {R_{h}}^{2}\cdot h\left(Fout_{f}\right)+{R_{h}}^{2}\cdot h\left(Fout_{e}\right) \end{array},$$

The torque and stiffness control in this article can be divided into two parts. The first part is the output force distribution of the mechanism on both the flexion and extension sides, and the second part is the calculation of the expected angle of the motor on both the flexion and extension sides. In the first part, combining Formulas (15) and (20), the output forces, $Fout_{e}$ and $Fout_{f}$, allocated to the extension and flexion sides are obtained based on the given expected torque $T_{ref}$ and stiffness $K_{ref}$. In the second part, the motors on both the extension and flexion sides are controlled to accurately output the force $Fout_{e}$ and $Fout_{f}$ obtained in the first part. The traditional series elastic actuator estimates the actual output force based on the deformation of the elastic body and uses it as feedback for control. For the variable stiffness actuators, stiffness is nonlinear, and this traditional control method introduces nonlinearity into the feedback loop, thereby affecting the control effect. Therefore, this article places stiffness with nonlinearity outside of the closed-loop control—that is, it converts the output force command Fout into the motor displacement command and performs closed-loop control on the motor displacement. This method does not introduce additional force sensors, and the output force of the variable stiffness actuator can be controlled through the built-in encoder of the motor and the encoder at the joint.

Combining Formulas (18) and (3), the deformation variable ΔL of the variable stiffness mechanism can be expressed as

(21) $$\left\{\begin{array}{l} \Delta L_{f}=I\left(\theta_{f}\right)=I\left[f^{-1}\left(Fout_{f}\right)\right]=J\left(Fout_{f}\right)\\ \Delta L_{e}==I\left(\theta_{e}\right)=I\left[f^{-1}\left(Fout_{e}\right)\right]=J\left(Fout_{e}\right) \end{array}\right.,$$

where I is the function of ΔL about θ, which is equivalent to formula (3), and J is the function of ΔL about Fout, which can be obtained by introducing the inverse function $f^{-1}$ into the function I.

Finally, by bringing Formula (21) into Formula (1), the expected rotation angle of the motor can be obtained as

(22) $$\left\{\begin{array}{l} \theta m_{f}=\frac{\Delta L_{f}}{R_{m}}+\frac{R_{h}\cdot \theta h}{R_{m}}=\frac{J\left(Fout_{f}\right)}{R_{m}}+\frac{R_{h}\cdot \theta h}{R_{m}}\\ \theta m_{e}=\frac{\Delta L_{e}}{R_{m}}-\frac{R_{h}\cdot \theta h}{R_{m}}=\frac{J\left(Fout_{e}\right)}{R_{m}}-\frac{R_{h}\cdot \theta h}{R_{m}} \end{array}\right.,$$

where $\theta m_{f}$ and $\theta m_{e}$ are the expected rotation angles of the hip flexion motor and the hip extension motor, respectively.

**Figure 6:**
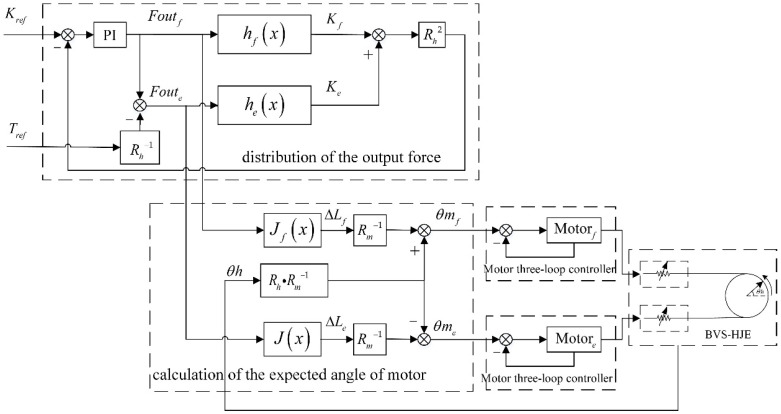
Control algorithm diagram of the BVS-HJE.

Formula (22) converts the output force obtained from the first part of the control strategy into a displacement command for the motor. Then, independent control of the exoskeleton output torque and stiffness can be achieved by implementing displacement closed-loop control on both motors separately. It should be noted that in the first part of the control strategy, Formulas (15) and (20) are nonlinear equations containing two independent variables and two dependent variables, and obtaining their analytical solutions is relatively difficult. Considering that PI control is a widely used and simple form of control, it is used to solve these equations in this article. Stiffness $K_{ref}$ is taken as the expected value, and Formulas (19) and (20) are considered as the descriptive equation for the controlled object. $Fout_{e}$ is expressed using $Fout_{f}$ based on Formula (16), and PI control is executed in this system. The control signal of the system is $Fout_{f}$, and $Fout_{e}$ can be obtained through Formula (16). In summary, a control algorithm diagram of the BVS-HJE is shown in Figure 6. The distribution of the output force in Figure 6 corresponds to the first part of the aforementioned control strategy, and the calculation of the expected angle of the motor corresponds to the second part. The three-loop controller of the motor in Figure 6, from the outside to the inside, consists of a position loop, speed loop, and current loop in sequence.

Given the above, it can be seen that the sensors in this study can be divided into two types. One type is an encoder installed at the hip joint, which is used to collect human motion information. Another type is the encoder installed in the variable stiffness unit, which is adopted to accurately estimate the output force and stiffness by collecting the angle information of the variable stiffness unit. The performance of the encoder at the hip joint directly affects the accuracy of human motion information collection under different motion postures. The encoder at the variable stiffness unit acts as an output sensor and is insensitive to changes in motion posture. In the future, IMU can be added to obtain more comprehensive and accurate human motion information.

## 4. Results

### 4.1. Simulation Results

**Figure 7:**
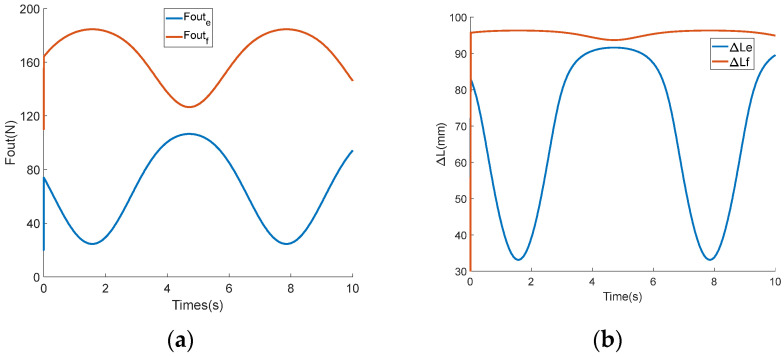
The simulation results. (**a**) Distribution of the output force; (**b**) calculation of the deformation variables.

**Figure 8:**
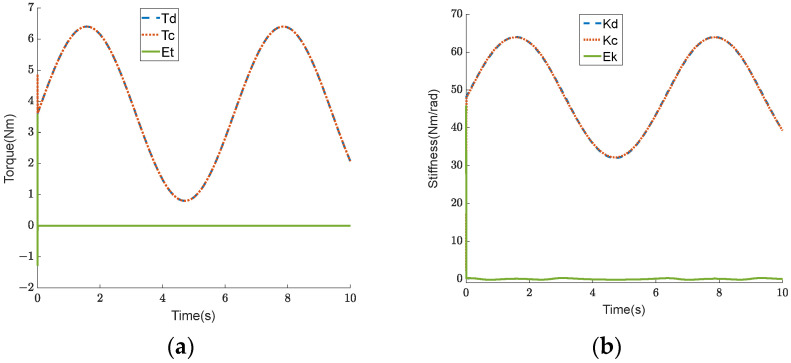
The simulation results. (**a**) Comparison curves for the output torque; (**b**) comparison curves for the output stiffness.

In order to verify the feasibility of the control strategy for coordinating the output force of the mechanism on both the flexion and extension sides, a simulation was performed and is detailed in this section. In the simulation, the friction coefficient μ and the hip joint rotation angle θh were both set to 0. The expected torque $T_{ref}$ was set to a sine curve with a bias of 3.6 Nm and an amplitude of 2.8 Nm, while the expected stiffness $K_{ref}$ was set to a sine curve with a bias of 48 Nm/rad and an amplitude of 16 Nm/rad. The output forces $Fout_{f}$ and $Fout_{e}$, allocated to both the flexion and extension sides in the first part of the control strategy, are shown in Figure 7a. The deformation variables $\Delta L_{f}$ and $\Delta L_{e}$, calculated from the output force in the second part of the control strategy, are shown in Figure 7b. A comparison of the curves between the output torque of the BVS-HJE caused by deformation variables and the expected torque is shown in Figure 8a, and a comparison of the curves between the output stiffness and the expected stiffness is shown in Figure 8b. The blue curve represents the expected value, the red curve represents the calculated value based on the control strategy, and the green curve represents the difference between the two. It can be seen that the error in torque is almost zero, and the maximum error in stiffness is 0.27 Nm/rad. The simulation results show that the control strategy proposed in this paper can convert the control of torque and stiffness into the control of deformation variables on both sides of the BVS-HJE, thereby achieving simultaneous control of the torque and stiffness.

### 4.2. Test Setup

**Figure 9:**
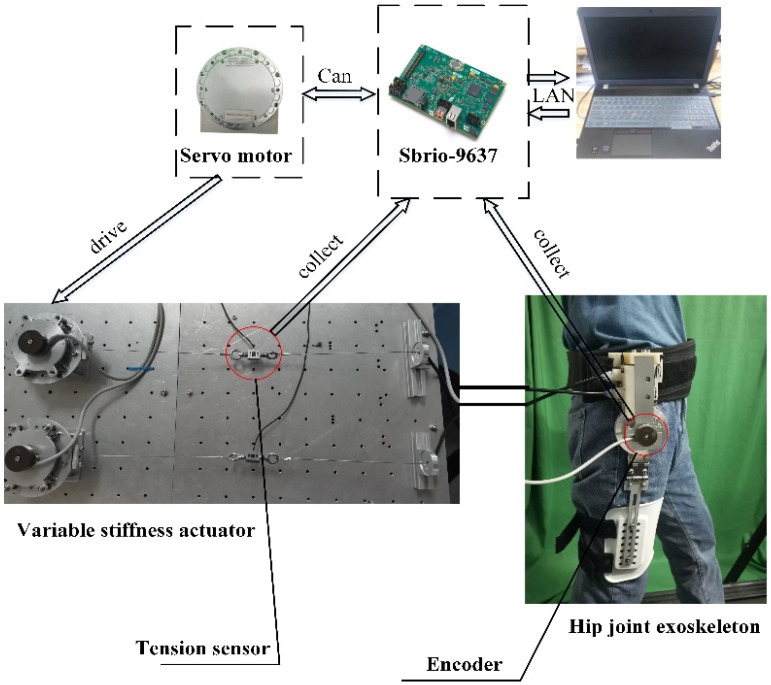
The designed BVS-HJE system.

Figure 9 shows the composition of the BVS-HJE system. The designed hip exoskeleton system consists of a mechanical part, a control part, and sensors. The mechanical part includes a variable stiffness actuator that mimics the antagonistic arrangement of human muscles, a hip joint body mechanism, and servo motors. The servo motors are internally integrated with an encoder and a reducer, with a reduction ratio of 6, an output-rated torque of 7.6 Nm, and a rated speed of 250 rpm. The control part consists of a host computer and a lower computer. The host computer is a personal computer in which the control program is written, compiled, and downloaded to the lower computer through LabVIEW. The lower computer is a Sbrio-9637, which is mainly responsible for receiving signals collected by sensors and controlling the motor. Sensors include encoders and tension sensors. Both the variable stiffness actuator and the hip exoskeleton are equipped with encoders, which are used to measure the rotation angles of the variable stiffness mechanism and the human hip joint, respectively. The tension sensor is located at the output of the variable stiffness mechanism and is used to measure the output force. This study was conducted in accordance with the Declaration of Helsinki, and the protocol of Registering Clinical Trials (ChiECRCT20200319) was approved by the Chinese Ethics Committee. In addition, the experimenter agreed to participate in the experiment plan.

**Figure 10:**
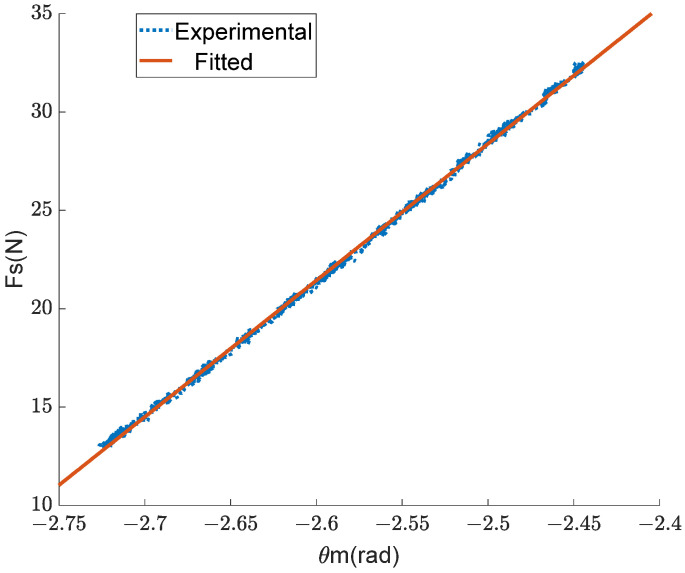
The results of the stiffness coefficient measurement experiment.

It was necessary to measure the stiffness coefficient of a single spring before conducting the experiment. One end of the spring is connected to the tension sensor and fixed on the table, while the other end is connected to a servo motor. The motor slowly reciprocates and records the motor angle and tension sensor data. The experimental results and fitted curve are shown in Figure 10.

The fitted equation is obtained as follows:

(23) $$\begin{array}{ccc} Fs & = & 69.44\theta m+202\\ & = & Ks\left(R_{m}\theta m-R_{m}\theta m_{0}\right) \end{array},$$

The R-squared value of the fitting result is 0.9991, indicating a close correlation between the fitting result and the experimental result. The radius of the motor winding wheel $R_{m}$ is 0.02 m, and the spring stiffness Ks was calculated as 3472 Nm based on Formula (23).

### 4.3. Verification Experiment on the Variable Stiffness Characteristics of the Mechanism

#### 4.3.1. Experiment in Which the Output End of the Mechanism Is Fixed

In the third section, a mathematical model of the variable stiffness mechanism was derived. In order to verify the variable stiffness characteristics of the mechanism proposed in this article, a reciprocating force-loading experiment in an ideal scenario was first adopted. In this experiment, we were concerned with studying the properties of the single-variable stiffness mechanism. Therefore, only one motor and one variable stiffness mechanism were used in the experimental setup. The output end of the mechanism was connected to the fixed end in the experiment to achieve the goal of eliminating the influence of human movement. The cable in the input end of the variable stiffness mechanism was connected to the motor. In terms of control, the motor was only placed in the speed loop to achieve reciprocating force loading by controlling the motor’s speed.

**Figure 11:**
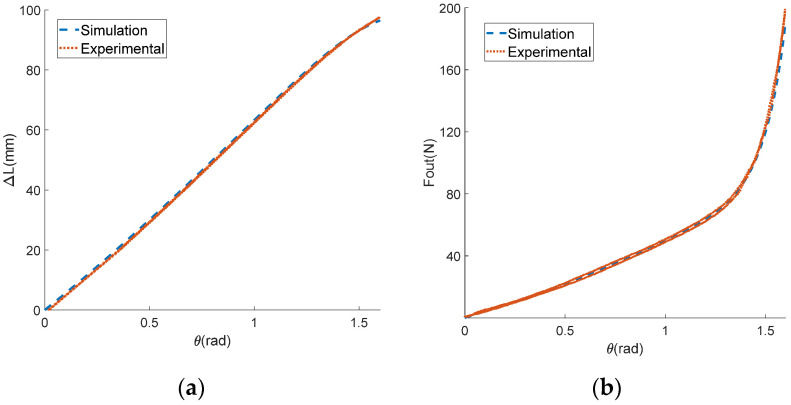
The experimental results of the reciprocating force loading experiment. (**a**) The relationship between the deformation variable and the mechanism rotation angle; (**b**) the relationship between the output force and the mechanism rotation angle.

**Figure 12:**
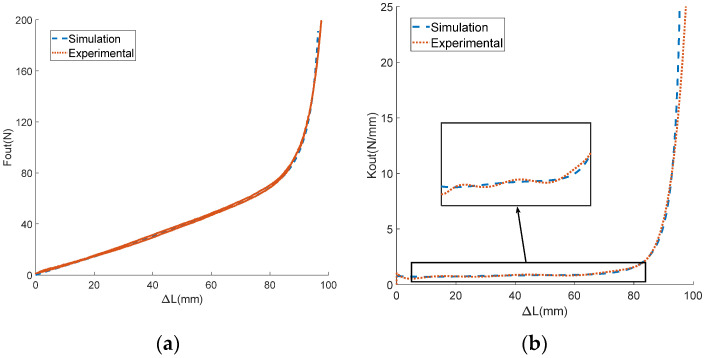
The experimental results of the reciprocating force loading experiment. (**a**) The relationship between the output force and the deformation variable; (**b**) the relationship between the output stiffness and the mechanism rotation angle.

In this experiment, the motor was first set to move back and forth at speeds of 1, 2, and 5 rad/s. To prevent the stroke being exceeded, the mechanism rotation angle θ was limited to 0–1.6 rad. The motor angle θm could be obtained by integrating the motor speed with time, and the displacement of the input end Xin could be obtained by multiplying the motor rotation angle by the groove radius. The output end was fixed with the displacement Xin=0. The encoder collected the actual rotation angle θ of the variable stiffness mechanism, while the tension sensor collected the actual output force Fout. The displacement difference between the input and output ends of the mechanism was the deformation variable ΔL. The experimental data on the output force and the deformation variable of the mechanism were processed, allowing us to obtain the derivative of the output force relative to the deformation variable, which was taken as the output stiffness Kout of the mechanism. The curves of ΔL with respect to θ, Fout with respect to θ, and Fout with respect to ΔL are shown in Figure 11a,b and Figure 12a, respectively. The curves of the actual and theoretical value of institutional stiffness are shown in Figure 12b. As a comparison, the corresponding curves of the theoretical model (due to the small friction force, the friction coefficient μ was set to 0) are also shown in the same figure as the experimental curves.

In Figure 11 and Figure 12, it can be seen that the experimental curve and the theoretical curve have the same trend of change. The error of the angle–displacement curve is within the range of [−2,2] mm, with the maximum error accounting for less than 2% of the theoretical curve. The maximum error of the angle–force curve is about 16 N, accounting for 8% of the theoretical value. The error of displacement–force curve does not exceed 9 N, which is only 4% of the theoretical value. The results of the reciprocating loading experiment demonstrate the accuracy of the theoretical model. In the experimental curve in Figure 12b, it can be seen that the stiffness of the mechanism changes with the variation in deformation, proving that the mechanism can achieve variable stiffness. According to Figure 12, it can be seen that the output force and stiffness of a single mechanism are related to the deformation variable. This proves the coupling characteristics of force and stiffness proposed in the theoretical analysis, and for a single-variable stiffness mechanism, it is difficult to simultaneously constrain the output force and stiffness.

#### 4.3.2. Experiment in Which the Output End of the Mechanism Is Fixed on the Hip Exoskeleton with Movement

The above experiment was conducted under ideal conditions. Considering situations that are closer to practical applications, the output end of the variable stiffness mechanism in the above experiment was changed to be connected to the hip joint exoskeleton. The tester put on hip exoskeletons with unilateral variable stiffness actuators and slowly swung their leg. Compensation for leg movement was determined by measuring the rotation angle of the hip exoskeleton through an encoder. In terms of control, different speeds (1, 2, and 5 rad/s) were still set to make the motor move back and forth.

**Figure 13:**
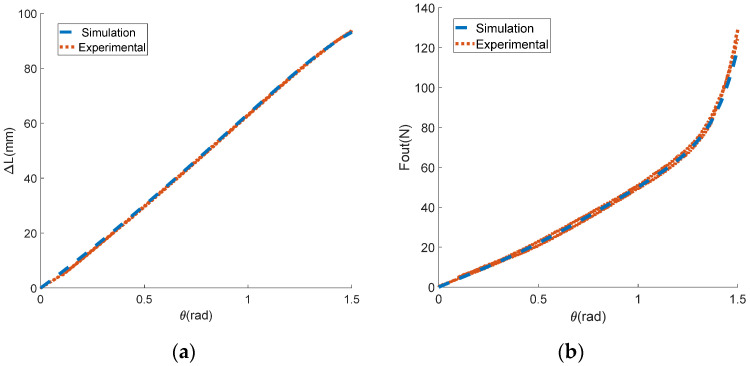
The results of the experiment with a human. (**a**) The relationship between the deformation variable and the mechanism rotation angle; (**b**) the relationship between the output force and the mechanism rotation angle.

**Figure 14:**
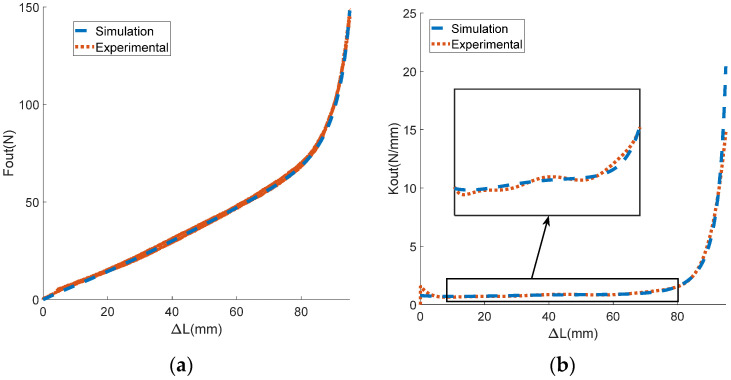
The results of the experiment with a human. (**a**) The relationship between the output force and the deformation variable; (**b**) the relationship between the output stiffness and the mechanism rotation angle.

Unlike in the previous experiments, the displacement on the output side of the variable stiffness mechanism was no longer 0 but rather corresponded to the displacement of the leg, which could be obtained by multiplying the angle of the encoder at the hip joint by the radius of the exoskeleton groove wheel. The deformation variable ΔL of the variable stiffness mechanism in this experiment is the difference between the displacement of the input side drive motor and the displacement of the leg, and the output force, Fout, of the mechanism was still measured by the force sensor. The curves of the relationship between Fout, ΔL, and θ are shown in Figure 13 and Figure 14. As a comparison, the corresponding theoretical curves are also shown in the same graph. The output force was derived from the deformation variable, and the result was used as the output stiffness of the mechanism. The curves of the actual and theoretical value of institutional stiffness are also shown in Figure 14b.

It can be seen that there is a significant similarity between the output force curves of the experiment with human intervention and the theoretical analysis in Figure 13, and it can be seen that the stiffness curve also has great consistency in Figure 14. Therefore, compensating for human motion through hip joint rotation has a certain level of viability.

However, there may be delays and measurement errors when using encoders to measure human motion angles. In other words, under the experimental conditions, having one end fixed can better characterize the properties of variable stiffness actuators. Therefore, the output force and stiffness curves of the variable stiffness actuator with a fixed output end are regarded as standard curves in this study, and the following controls are based on this.

### 4.4. Verification Experiment of the Coordinated Control of the Output Torque and Stiffness of the BVS-HJE

**Figure 15:**
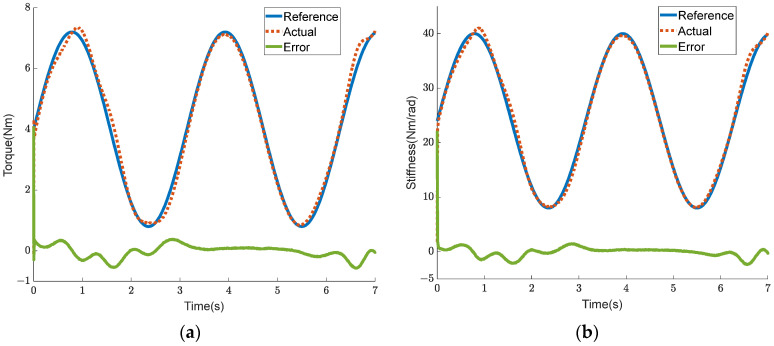
The dynamic curves under the coordinated control of the BVS-HJE. (**a**) The dynamic curve of the torque response; (**b**) the joint stiffness.

**Figure 16:**
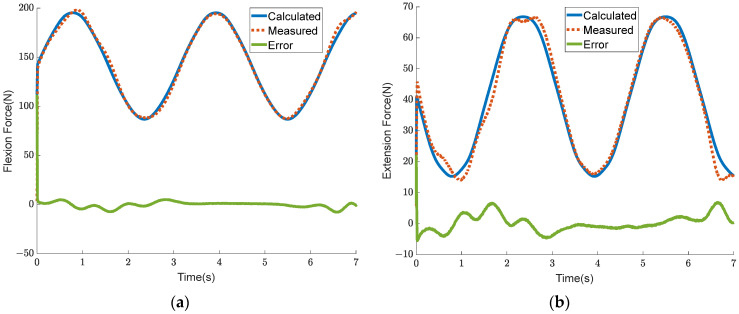
Experimental results of the output force of the variable stiffness actuators on both the flexion and extension sides. (**a**) Flexion force; (**b**) extension force.

The human body has different requirements for joint torque and joint stiffness under different working conditions. When the human body is standing still, the hip joint requires greater stiffness to ensure stability, while the torque requirement is zero. During leg swinging, a high auxiliary torque and low stiffness are required. In a squatting state, a certain degree of auxiliary torque and auxiliary stiffness are required simultaneously. It is also crucial to achieve both torque and stiffness control in practical applications. In order to verify that the BVS-HJE can coordinate the control of output torque and stiffness, an experiment was conducted. The expected torque $T_{ref}$ was set to a sine curve with a bias of 4 Nm and an amplitude of 3.2 Nm, while the expected stiffness $K_{ref}$ was set to a sine curve with a bias of 24 Nm/rad and an amplitude of 16 Nm/rad. The experimental results are shown in Figure 15 and Figure 16. Figure 15a, b show the torque tracking curve and joint stiffness tracking curve, respectively. In Figure 15, it can be seen that the torque tracking error is less than 0.7 Nm and the stiffness tracking error is less than 2.5 Nm/rad, proving that the proposed strategy can simultaneously control the output torque and stiffness of the BVS-HJE. Figure 16 shows the calculated output force that should be allocated to both the flexion and extension sides in the control strategy of this article, as well as the actual measured output force. The force errors are all less than 10 N, indicating the effectiveness of the control strategy in achieving output force control by adjusting the motor angle. It is worth noting that there is still room for improvement in the accuracy of the output force control, which may be due to the imprecise mathematical model of the mechanism and human motion.

### 4.5. Experiment on Assisted Leg Swing with Passive Compliant Control

The previous section evaluated the independent control function of joint torque and stiffness of BVS-HJE. The performance of this system in passive compliant control is equally important. Unlike torque control, passive control generates a reference trajectory and moves the wearer’s leg along this trajectory. Therefore, the position control of the joints is regarded as the outermost loop, and the error between the reference joint trajectory and the actual joint trajectory is modulated by the PI controller to generate the reference torque of the inner loop controller. The inner loop of the controller is the same as the previous section, consisting of the torque control loop and the stiffness control loop. In the experiment, the expected joint trajectory is set as a sine signal with an amplitude of 20 degrees and a frequency of 0.25 Hz, and the expected joint stiffness is set as a linear function with a slope of 1.5 and an offset of 10.

**Figure 17:**
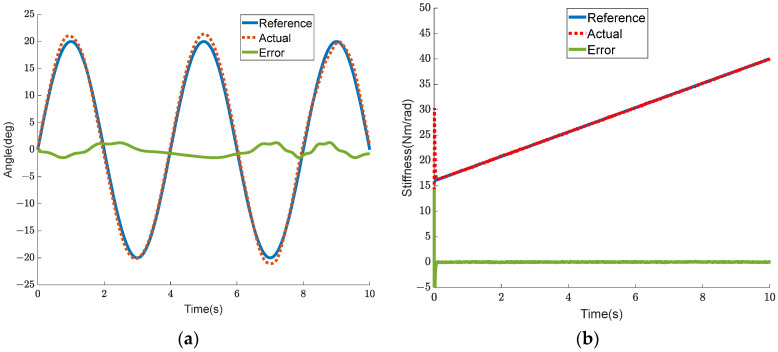
Experiment results of passive compliance control. (**a**) Tracking curve of the joint trajectory; (**b**) the curve of the joint stiffness.

**Figure 18:**
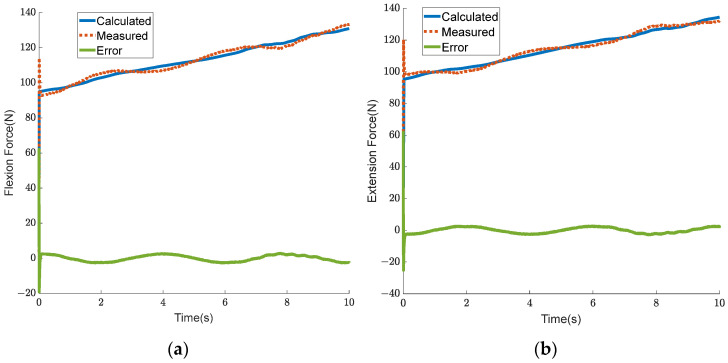
Experimental results of the output force for passive compliance control on both the flexion and extension sides. (**a**) Flexion force; (**b**) extension force.

The experimental results are shown in Figure 17 and Figure 18. Figure 17a,b show the joint trajectory tracking curve and joint stiffness tracking curve, respectively. In Figure 17a, it can be seen that the angle tracking error is less than 1.5 deg. In Figure 17b, the stiffness trajectory closely matches the reference one, and the tracking error is almost zero. This proves that independent stiffness control can also be achieved in passive trajectory tracking of the BVS-HJE. Figure 18 shows the calculated output force that should be allocated to both the flexion and extension sides in the control strategy, as well as the actual measured output force. The force errors are all less than 5 N, indicating the effectiveness of the control strategy in achieving output force control by adjusting the motor angle.

## 5. Conclusions

This article presents a biomimetic variable stiffness hip joint exoskeleton (BVS-HJE). By arranging the variable stiffness mechanisms antagonistically on the hip flexion and extension sides, the exoskeleton was able to simultaneously control joint stiffness and torque, similar to the human body. A single variable stiffness mechanism model was established, and the theoretical relationship between the deformation variables of the mechanism and output force and stiffness was obtained. In addition, based on the characteristics of the BVS-HJE, a control strategy was proposed that can achieve independent adjustment of joint torque and joint stiffness. The mechanism’s variable stiffness property was verified through reciprocating loading tests. Comparing experimental and theoretical data, both curves had the same change trend, which, to some extent, verified the correctness of the theoretical model. Through torque control and stiffness control experiments using the hip joint exoskeleton, it was verified that the BVS-HJE could simultaneously achieve independent control of torque and stiffness. The developed BVS-HJE is compliant and can adjust stiffness while controlling torque or position. It should be noted that the BVS-HJE also has some limitations. Due to the overly compact design of the mechanism, the selected pulley radius is relatively small. This may cause the Bowden cable to slip out of the pulley groove and get stuck in the gap of the mechanism, thereby increasing friction loss and affecting the control effect. The structure of the device still has room for improvement. During the active assistance process, the output force and output stiffness can be controlled simultaneously according to the user’s wishes, improving human–machine interaction performance. Passive control can be used in rehabilitation medicine to adjust the output stiffness during motion tracking so as to meet users’ needs for different impedances. Methods for selecting appropriate torque and stiffness reference curves were not provided in this article. This involves upper- or middle-level control strategies and will be the focus of future research.

## Figures and Tables

**Table 1:** Mechanical parameters of the device.

Parameters	Value (m)
$R_{m}$	0.02
$R_{h}$	0.04
R	0.007
Ra	0.027
Rb	0.045
E	0.018
Rsr	0.015
Rsf	0.045

## Data Availability

Data are contained within the article.

## References

[1] Sado F., Yap H.J., Ghazilla R.A.R., Ahmad N.B. (2019). Design and control of a wearable lower-body exoskeleton for squatting and walking assistance in manual handling works. Mechatronics.

[2] Coenen P., Gouttebarge V., van der Burght A.S., van Dieën J.H., Frings-Dresen M.H., van der Beek A.J., Burdorf A. (2014). The effect of lifting during work on low back pain: A health impact assessment based on a meta-analysis. Occup. Environ. Med..

[3] Santos C., Gabriel A.T., Quaresma C., Nunes I.L., Arezes P.M., Melo R.B., Carneiro P., Branco J.C., Colim C.A., Costa N., Costa S., Duarte J., Guedes J.C., Perestrelo G. (2023). Risk Factors for Lower Limb Work-Related Musculoskeletal Disorders. Occupational and Environmental Safety and Health V.

[4] Sasaki M., Horio A., Wakasa M., Uemura S., Osawa Y. (2008). Influence of quadriceps femoris fatigue on low back load during lifting of loads at different distances from the toes. J. Phys. Ther. Sci..

[5] Chen B., Zhong C.H., Zhao X., Ma H., Guan X., Li X., Liang F.Y., Cheng J.C.Y., Qin L., Law S.W. (2017). A wearable exoskeleton suit for motion assistance to paralysed patients. J. Orthop. Transl..

[6] Liu J., He Y., Yang J., Cao W., Wu X. (2022). Design and analysis of a novel 12-dof self-balancing lower extremity exoskeleton for walking assistance. Mech. Mach. Theory.

[7] Gao M., Wang Z., Pang Z., Sun J., Li J., Li S., Zhang H. (2022). Electrically Driven Lower Limb Exoskeleton Rehabilitation Robot Based on Anthropomorphic Design. Machines.

[8] Li J.F., Cao Q., Dong M.J., Zhang C.Z. (2021). Compatibility evaluation of a 4-DOF ergonomic exoskeleton for upper limb rehabilitation. Mech. Mach. Theory.

[9] McCann C.M., Hohimer C.J., O’Neill C.T., Young H.T., Bertoldi K., Walsh C.J. (2023). In-situ measurement of multi-Axis torques applied by wearable soft robots for shoulder assistance. IEEE Trans. Med. Robot. Bionics.

[10] Gull M.A., Thoegersen M., Bengtson S.H., Mohammadi M., Andreasen Struijk L.N.S., Moeslund T.B., Bak T., Bai S. (2021). A 4-DOF Upper Limb Exoskeleton for Physical Assistance: Design, Modeling, Control and Performance Evaluation. Appl. Sci..

[11] Fang Q., Li G., Xu T., Zhao J., Cai H., Zhu Y. (2019). A Simplified Inverse Dynamics Modelling Method for a Novel Rehabilitation Exoskeleton with Parallel Joints and Its Application to Trajectory Tracking. Math. Probl. Eng..

[12] Zhang F., Lin L., Yang L., Fu Y. (2019). Design of an Active and Passive Control System of Hand Exoskeleton for Rehabilitation. Appl. Sci..

[13] Wang Y., Wang H.P., Tian Y. (2022). Adaptive interaction torque-based AAN control for lower limb rehabilitation exoskeleton. ISA Trans..

[14] Meng W., Liu Q., Zhou Z., Ai Q., Sheng B., Xie S.S. (2015). Recent development of mechanisms and control strategies for robot-assisted lower limb rehabilitation. Mechatronics.

[15] Vanderborght B., Albu-Schäffer A., Bicchi A., Burdet E., Caldwell D.G., Carloni R., Catalano M., Eiberger O., Friedl W., Ganesh G. (2013). Variable impedance actuators: A review. Robot. Auton. Syst..

[16] Rodriguez-Cianca D., Weckx M., Jimenez-Fabian R., Torricelli D., Gonzalez-Vargas J., Sanchez-Villamañan M.C., Sartori M., Berns K., Vanderborght B., Pons J.L. (2019). A Variable Stiffness Actuator Module With Favorable Mass Distribution for a Bio-inspired Biped Robot. Front. Neurorobotics.

[17] Li Z., Huang B., Ye Z., Deng M., Yang C. (2018). Physical Human-Robot Interaction of a Robotic Exoskeleton By Admittance Control. IEEE Trans. Ind. Electron..

[18] Yan Y., Chen Z., Huang C., Chen L., Guo Q. (2022). Human-exoskeleton coupling dynamics in the swing of lower limb. Appl. Math. Model..

[19] Chen Z., Guo Q., Li T., Yan Y. (2023). Output Constrained Control of Lower Limb Exoskeleton Based on Knee Motion Probabilistic Model With Finite-Time Extended State Observer. IEEE/ASME Trans. Mechatron..

[20] Luo R.M., Sun S.Q., Zhao X.Y., Zhang Y.X., Tang Y. Adaptive CPG-based impedance control for assistive lower limb exoskeleton. Proceedings of the 2018 IEEE International Conference on Robotics and Biomimetics (ROBIO).

[21] Hsieh H.C., Chen D.F., Chien L., Lan C.C. (2017). Design of a Parallel Actuated Exoskeleton for Adaptive and Safe Robotic Shoulder Rehabilitation. IEEE-ASME Trans. Mechatron..

[22] Ando K., Hirokawa M., Suzuki K. Fusion of musculoskeletal and dynamic models to estimate knee joint impedance for exoskeleton control. Proceedings of the 2023 IEEE/SICE International Symposium on System Integration (SII).

[23] Qu Z., Wei W., Wang W., Zha S., Li T., Gu J., Yue C. Research on fuzzy adaptive impedance control of lower extremity exoskeleton. Proceedings of the 2019 IEEE Int. Conf. on Mechatronics and Automation (ICMA).

[24] An M.L., Wang X.J., Miao Y.A., Wang S.P., Miao Y.Q. Trajectory design and adaptive impedance control of lower limb exoskeleton. Proceedings of the 16th IEEE Conference on Industrial Electronics and Applications (ICIEA).

[25] Migliore S.A., Brown E.A., Deweerth S.P. Biologically Inspired Joint Stiffness Control. Proceedings of the 2005 IEEE International Conference on Robotics and Automation (ICRA).

[26] Wolf S., Grioli G., Eiberger O., Friedl W., Grebenstein M., Höppner H., Burdet E., Caldwell D.G., Carloni R., Catalano M.G. (2016). Variable stiffness actuators: Review on design and components. IEEE/ASME Trans. Mechatron..

[27] Pott P.P., Müller R., Grun M., Konigorski U., Schlaak H.F. (2013). Serial-elastic actuators for active orthoses. At-Automatisierungstechnik.

[28] Herbin P., Pajor M. (2021). Human-robot cooperative control system based on serial elastic actuator bowden cable drive in ExoArm 7-DOF upper extremity exoskeleton. Mech. Mach. Theory.

[29] Bergmann L., Voss D., Leonhardt S., Ngo C. (2023). Lower-Limb Exoskeleton With Compliant Actuators: Human Cooperative Control. IEEE Trans. Med. Robot. Bionics.

[30] Hollander K.W., Sugar T.G., Herring D.E. Adjustable robotic tendon using a ‘Jack Spring’. Proceedings of the 9th IEEE International Conference on Rehabilitation Robotics (ICORR).

[31] Morita T., Sugano S. Design and development of a new robot joint using a mechanical impedance adjuster. Proceedings of the 1995 IEEE International Conference on Robotics and Automation (ICRA).

[32] Li Z.Y., Bai S.P., Madsen O., Chen W.H., Zhang J.B. (2020). Design, modeling and testing of a compact variable stiffness mechanism for exoskeletons. Mech. Mach. Theory.

[33] Zhu Y., Wu Q., Chen B., Xu D., Shao Z. (2022). Design and Evaluation of a Novel Torque-Controllable Variable Stiffness Actuator with Reconfigurability. IEEE/ASME Trans. Mechatron..

[34] Zhu Y., Bai S.P. (2023). Human Compatible Stiffness Modulation of a Novel VSA for Physical Human-Robot Interaction. IEEE Robot. Autom. Lett..

[35] Shao Y.X., Zhang W.X., Su Y.J., Ding X.L. (2021). Design and optimisation of load-adaptive actuator with variable stiffness for compact ankle exoskeleton. Mech. Mach. Theory.

[36] Hurst J.W., Rizzi A.A. (2008). Series compliance for an efficient running gait—Lessons learned from the electric cable differential leg. IEEE Robot. Autom. Mag..

[37] Tonietti G., Schiavi R., Bicchi A. Design and Control of a Variable Stiffness Actuator for Safe and Fast Physical Human/Robot Interaction. Proceedings of the 2005 IEEE International Conference on Robotics & Automation (ICRA).

[38] Kang G., Oh H.S., Seo J.K., Kim U., Choi H.R. (2019). Variable admittance control of robot manipulators based on human intention. IEEE/ASME Trans. Mechatron..

[39] Grosu V., Rodriguez-Guerrero C., Grosu S., Vanderborght B., Lefeber D. (2017). Design of smart modular variable stiffness actuators for robotic-assistive devices. IEEE/ASME Trans. Mechatron..

[40] Zhu Y., Wu Q., Chen B., Zhao Z. (2022). Design and Voluntary Control of Variable Stiffness Exoskeleton Based on sEMG Driven Model. IEEE Robot. Autom. Lett..

[41] Hogan N. (1984). Adaptive control of mechanical impedance by coactivation of antagonist muscles. IEEE Trans. Autom. Control.

